# **Coumarin compounds as fungicidal agents against powdery mildew and rust in cereals**

**DOI:** 10.1038/s41598-026-40869-w

**Published:** 2026-02-24

**Authors:** Klaudia Rząd, Aleksandra Nucia, Katarzyna Szwaczko, Arkadiusz Matwijczuk, Sylwia Okoń

**Affiliations:** 1 Department of Biophysics, Faculty of Environmental Biology, University of Life Sciences in Lublin, Akademicka 13, Lublin, 20-950 Poland; 2 Institute of Plant Genetics, Breeding and Biotechnology, University of Life Sciences in Lublin, Akademicka 15, Lublin, 20-950 Poland; 3 Department of Organic Chemistry and Crystallochemistry, Institute of Chemical Sciences, Faculty of Chemistry, Marie Curie-Skłodowska University in Lublin, 33 Gliniana, Lublin, Poland

**Keywords:** Blumeria, Puccinia, Agrochemicals, Coumarins, Agroecology, Fungal pathogenesis, Pathogens

## Abstract

Parasitism is a harmful relationship between microorganisms and their host. Biotrophic fungi, such as powdery mildew and rusts, are obligate parasites that extract nutrients from living plant cells using specialized structures like haustoria and appressoria. These pathogens weaken plant defenses by inhibiting enzyme secretion, making them particularly dangerous. *Blumeria graminis*, causing powdery mildew, affects all cereals and forms a white coating. *Pucciniales*, or rusts, represent the largest group of biotrophs, with complex life cycles involving up to five spore stages, complicating their identification. Resistance to chemical pesticides and environmental concerns have driven interest in alternative, safer, plant protection solutions. One promising group is coumarin and its derivatives, known for antifungal properties and biodegradability. Our study aimed to test the effectiveness of selected coumarin compounds in inhibiting the growth of powdery mildew and rust under laboratory conditions. These findings may support the development of modern, eco-friendly fungicides. Our studies have shown that the tested coumarin derivatives were characterized by varying effectiveness in inhibiting the growth of biotrophic pathogenic fungi. Histological studies showed that two compounds led to a reduction in the number of pathogen structures, suggesting their potential effectiveness in inhibiting the spread of infection.

**Supplementary Information:**

The online version contains supplementary material available at 10.1038/s41598-026-40869-w.

##  Introduction

Microorganisms can engage in various interactions with their host, ranging from beneficial to harmful^1^. These interactions have been categorized into three classes: mutualism, commensalism, and parasitism^2^. Mutualism occurs when both interacting species benefit from the relationship. In commensalism, only one organism benefits from the association, while the other remains unaffected^3^. On the other hand, parasites colonize their host and extract trophic resources, often leading to damage, such as necrosis^4^. Parasitic fungi pose a significant threat to plants. After infecting the host, fungi may employ several strategies to obtain nutrients^5^. The first strategy is used by necrotrophs, which, upon attaching to the plant, rapidly kill its cells, becoming saprotrophs that feed on the dead plant tissue^6^. Another strategy is employed by biotrophs, which derive energy and nutrients from the living cells of the host^7,8^. Biotrophic fungi are characterized by highly developed infectious structures and haustoria, specialized hyphae for extracting nutrients from plant cells. Additionally, these fungi suppress the secretory activity of plants, particularly lytic enzymes, resulting in a prolonged suppression of the plant’s response to pathogen attack^9^. These properties make biotrophic fungi one of the most dangerous groups of pathogens in the world. Among biotrophs, the largest group of obligate pathogens includes fungi causing rusts and powdery mildews^10^.

*Blumeria graminis* is an obligate biotroph that causes a disease known as powdery mildew of cereals and grasses. This pathogen attacks all types of cereals, and the infection symptom is a characteristic white, powdery coating^11^. During asexual reproduction cycles, it produces large amounts of conidial spores, which are dispersed by the wind and contribute to the spread of the disease. The spores germinate on the surface of the leaves, penetrating the host’s epidermis to then form haustoria that extract nutrients^12,13^. The developing mycelium covers the leaf surface, negatively affecting the plant’s metabolism and photosynthesis process, reducing the number of panicles, the thousand-grain weight, and ultimately lowering biomass production. The onset of the disease also leads to a decrease in the protein content of the grain^14,15^.

It is believed that the most species-rich group of obligate biotrophs attacking cultivated plants are fungi of the order *Pucciniales*, which cause diseases known as rusts^16^. These are pathogenic organisms exhibiting significant complexity in both their hosts and life cycles^17^. Such a life cycle can include up to five stages of rust fungus spores, making the identification of rust fungi challenging^18^. The pathogens appear on plants as a rusty powder formed from spores. *Pucciniales* hinder the photosynthesis process in plants. By reducing crop yields, these fungi cause both economic and ecological damage^19^.

Pathogens that cause damage to crops and reduce the quality and quantity of yields have forced the agricultural sector to use plant protection products. This is a crucial element in ensuring food security. However, there is a threat to the effectiveness of these products, namely the evolution of pathogens toward developing resistance^20^. Studies show that after the introduction of newly synthesized pesticides aimed at eliminating the threat posed by microorganisms on plants, fungi can rapidly develop resistance to chemical agents. As it turns out, pathogens can develop biochemical mechanisms that make them less sensitive to applied agrochemicals. These mechanisms include mutations, overexpression, and metabolic degradation, among others^21^. Furthermore, the intensive use of synthetic pesticides can have a negative impact on the environment^22^. These factors highlight the need to search for new, effective, and environmentally friendly plant protection measures. Currently, in the development of new agrochemical products, particular attention is being paid to natural substances. One such substance is coumarin and its derivatives^23^^,^^24^.

**Fig. 1:**
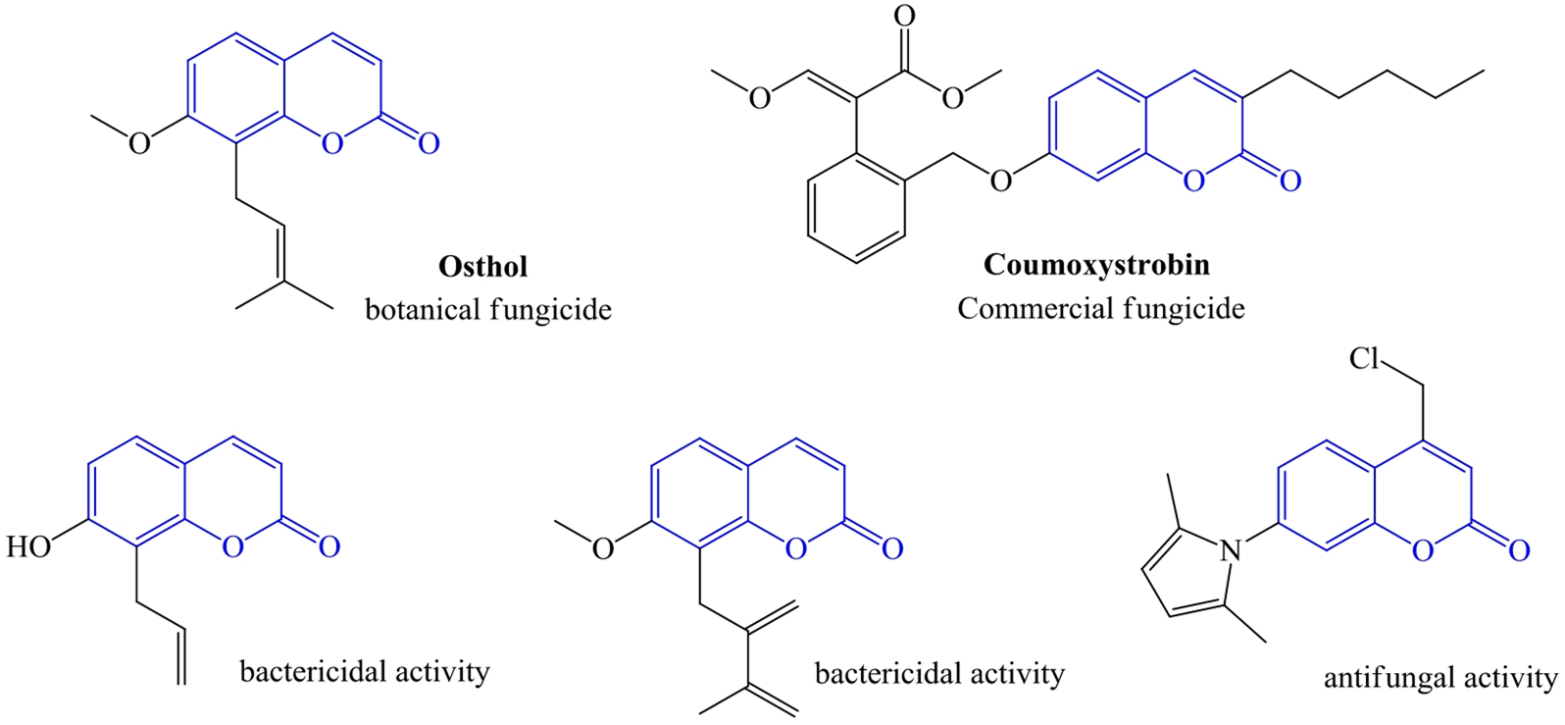
Antifungal and antibacterial coumarin derivatives.

Coumarins are a promising group of natural active compounds widely used in medicine^25–27^ and agriculture^28–30^. As plant protection agents, coumarins exhibit versatile biological activities, including fungicidal^31^, insecticidal^32^, antiviral^33^, and herbicidal properties^34^, Fig. 1. Due to their biological activity, compounds from the coumarins group are used in commercial plant protection products to combat cereal diseases. Their simple chemical structure and ability to modify their framework allow almost arbitrary introduction of functional groups, such as esters, amides, acyl, and other groups, into the coumarin skeleton, which enhance their biological activity and improve lipid solubility. Alternatively, chemicals with groups like allyl or vinyl have been shown to be able to bind to bacterial or fungal cells and stop them from working properly, which stops the growth of pathogens^23,35^.

**Fig. 2:**
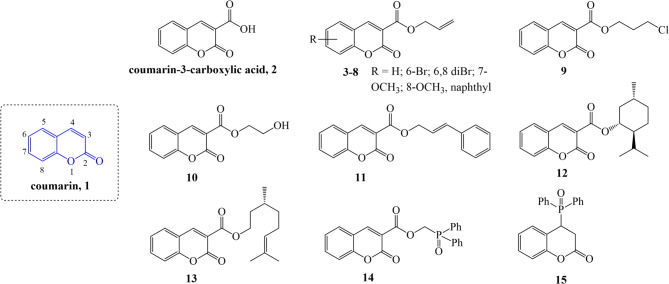
Coumarin and its derivatives evaluated in this work.

With this in mind, we aimed to synthesise allyl (**3–8**) and alkyl esters of coumarin-3-carboxylic acid (**9–14**) as well as the 4,5 -dihydrocoumarin derivative (**15**), a sub-class of coumarin (Fig. 2.). Coumarin (**1**) and coumarin-3-carboxylic acid (**2**) were employed as reference compounds. All compounds were tested for their ability to inhibit the growth and development of biotrophic fungal pathogens that induce cereal diseases. To our knowledge, the potential fungicidal activity of coumarin derivatives **3–15** has not been investigated to date, although compound **1** (natural coumarin) has previously been reported to show antifungal effects against *Botrytis cinerea*, *Fusarium graminearum*, *Magnaporthe oryzae*, *Sclerotinia sclerotiorum*, and *Rhizoctonia solani*^36^, and compound **2** (a carboxylated derivative) was studied in vitro for its antibacterial and antifungal activity against *Botrytis cinerea*, *Alternaria solani*, *Gibberella zeae*, *Rhizoctonia solani*, cucumber anthracnose, and *Alternaria* leaf spot^37^. However, such studies have not been conducted for biotrophic pathogens.

##  Results

### Synthesis of synthesis of coumarin derivatives for biological evaluation

Coumarin, (**1**, 2 H-1-benzopyran-2-one) was derived from commercial sources. Coumarin derivatives **2–15** were synthesized using established methods emphasizing simplicity, efficiency, and minimizing waste generation. Full ^1^H NMR, ^13^C NMR, ^31^P NMR, and mass spectroscopy analyses were used to confirm the structures and purity of all synthesized compounds. NMR spectra for the new compounds **7**, **9**, **13**, and **14** are included in the supplementary materials (Figure S1-S9).

Coumarin-3-carboxylic acid (**2**) was synthesized from Meldrum’s acid and salicylaldehyde employing potassium carbonate *via* Knoevenagel condensation, followed by intramolecular cyclization^38^. The resulting products were purified by crystallography with ethanol or column chromatography employing a mixture of hexane and ethyl acetate.

#### Synthetic strategies for the synthesis of compounds 3–15

Compounds **3–8** were synthesized by a simple Knoevenagel condensation reaction. This process is widely exploited in organic chemistry for the formation of new C-C bonds^39,40^. In its classical version, the reaction proceeds at elevated temperatures and requires the presence of catalytic amounts of organic bases, such as pyrrolidine, pyridine, or piperidine, and an acid, such as acetic acid.

**Fig. 3:**
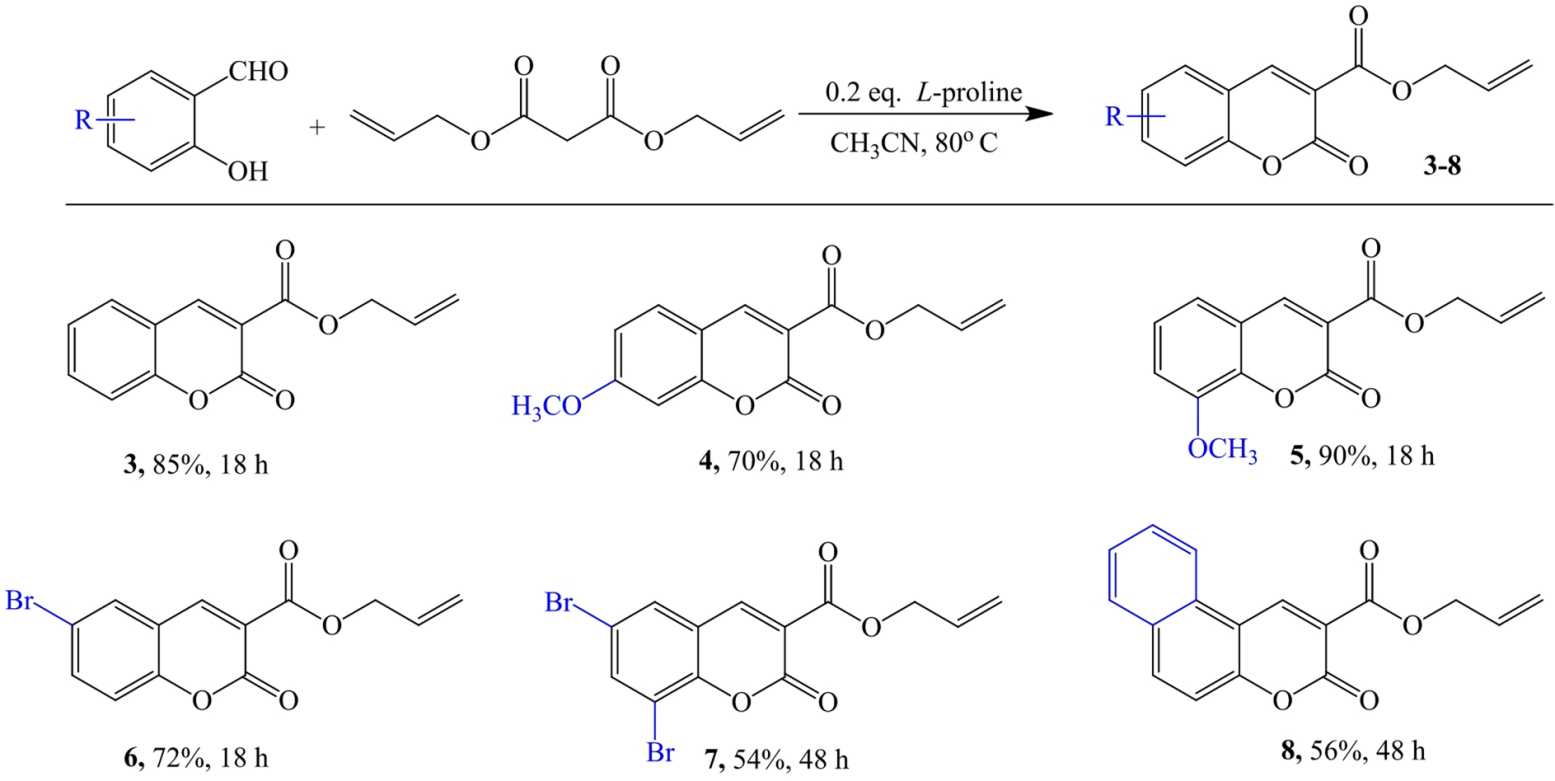
The synthesis of allyl esters of coumarin-3-carboxylic acid **3–8**.

In the preparation of coumarin derivatives **3–8**, these catalysts were successfully excluded, and a non-toxic organic amine was chosen to improve the environmental performance of the synthesis process. The amino acid *L*-proline at 0.2 eq. functioned as a single catalyst, therefore minimizing the use of potentially hazardous reactants (Fig. 3.). *L*-proline has a number of advantages that make it an attractive catalyst: (a) it is bifunctional, by which it can behave as both an acid and a base, (b) it is cheap and commercially available; (c) most importantly, it is natural and environmentally friendly. We therefore proceeded with a series of condensation reactions of diallyl malonate with salicylaldehyde derivatives. The reaction mixture was concentrated, and the target reaction products were derived by simple crystallization from ethanol. The pure reaction products were analyzed by NMR spectroscopy.

In the synthesis of compound **3**, diallyl malonate was subjected to Knoevenagel condensation with salicyl aldehyde in the presence of *L*-proline. At 80 °C in acetonitrile and after 18 h, the target coumarin was synthesized in very good yields of 85%. In an analogous manner, compounds **4–6** were afforded with very good yields (90 − 70%). To get coumarin esters **7** and **8**, it was necessary to extend the reaction time to 48 h; the yields were 54% and 56%, respectively.

**Fig. 4:**
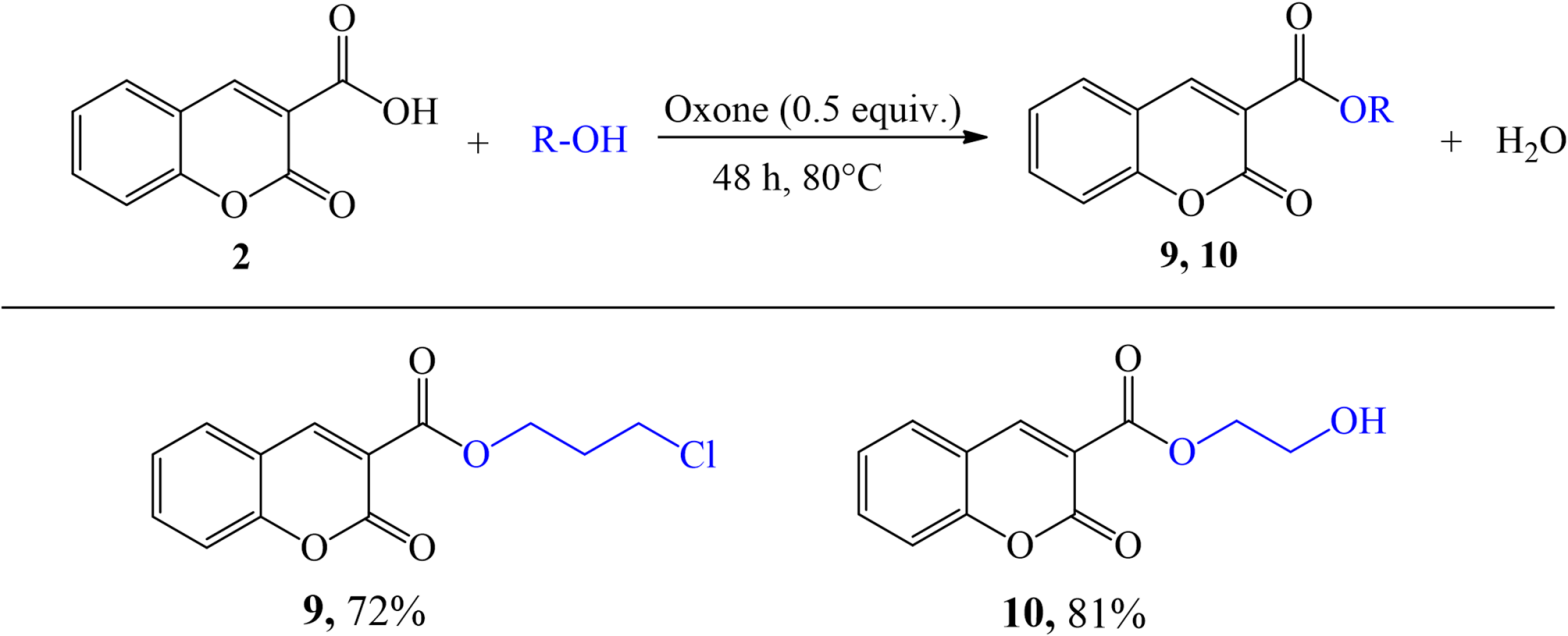
The esterification of coumarin-3-carboxylic acid with alcohols.

Coumarin derivatives **9** and **10** were prepared in very good yields (72% and 81%, respectively) on the esterification of coumarin-3-carboxylic acid (Fig. 4.). A non-toxic Oxone salt of 0.5 eq. was employed as a catalyst, which can be readily removed from the reaction mixture with a 5% NaHCO₃ solution. The reaction proceeded at 80 °C for 48 h in an appropriate alcohol (3-chloro-1-propanol or ethylene glycol) which acted as both reactant and solvent in the reaction.

**Fig. 5:**
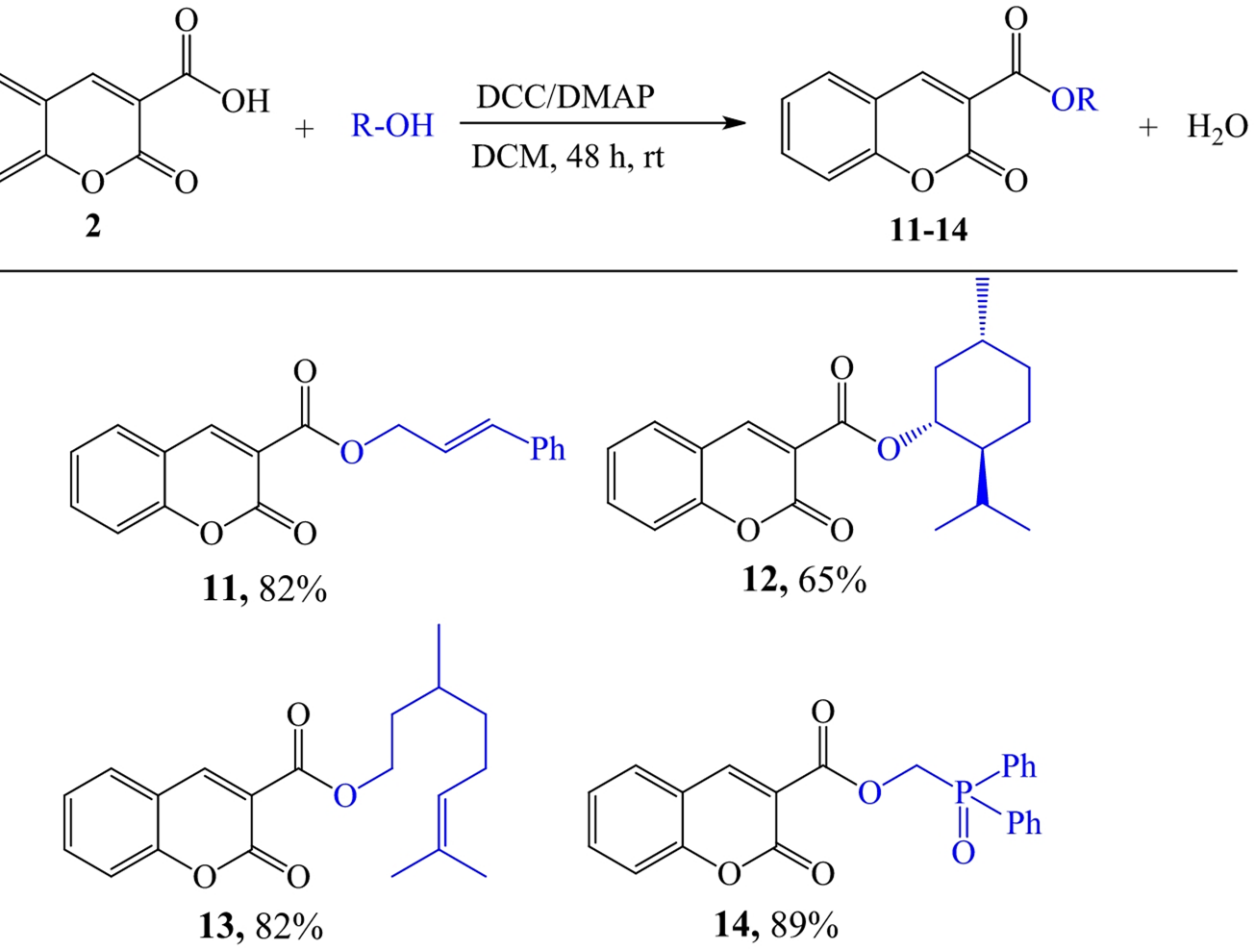
The synthesis of coumarins **11–14** *via* the Steglich esterification.

In the synthesis of coumarins **11–14** we applied the Steglich esterification, which was a more efficient approach (Fig. 5.). The esterification of coumarin-3-carboxylic acid (**2**) with diverse alcohols was carried out employing 1.1 equivalents of DCC (*N*,* N’*-dicyclohexyl-carbodiimide) and 0.05 equivalents of DMAP (4-dimethylaminopyridine), with dichloromethane (DCM) acting as the solvent. The reactions were maintained at room temperature for a period of 48 h. The resulting compounds were initially purified using column chromatography with a hexane/ethyl acetate mixture. The subsequent recrystallisation from ethyl alcohol produced coumarins **11–14** with yields ranging from 65% to 89%.

**Fig. 6:**
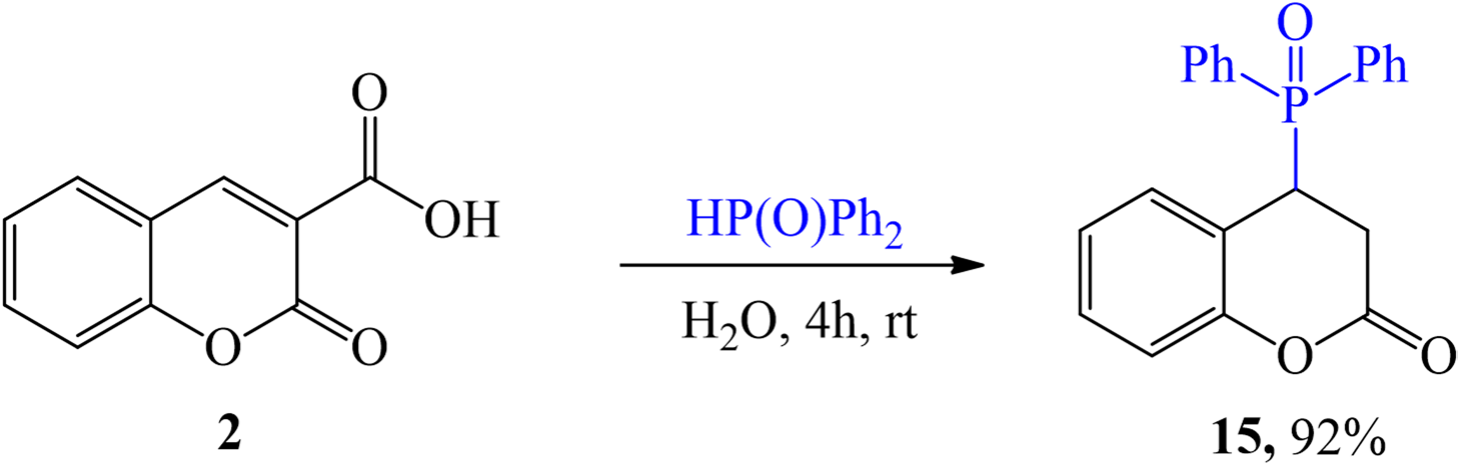
Synthetic route to the compound **15**.

The 4, 5-dihydrocoumarin **15** derivative was derived by direct phosphorylation of coumarin-3-carboxylic acid according to the procedure described by Brahmachari (Fig. 6.)^41^.

The reaction was carried out in water at rt., employing 1.0 eq. of diphenylphosphine oxide. After a time of 4 h, the target product was sufficiently filtered and dried to give derivative **15** in 92% yield.

### Evaluation of antifungal activity against biotrophic pathogens

**Fig. 7:**
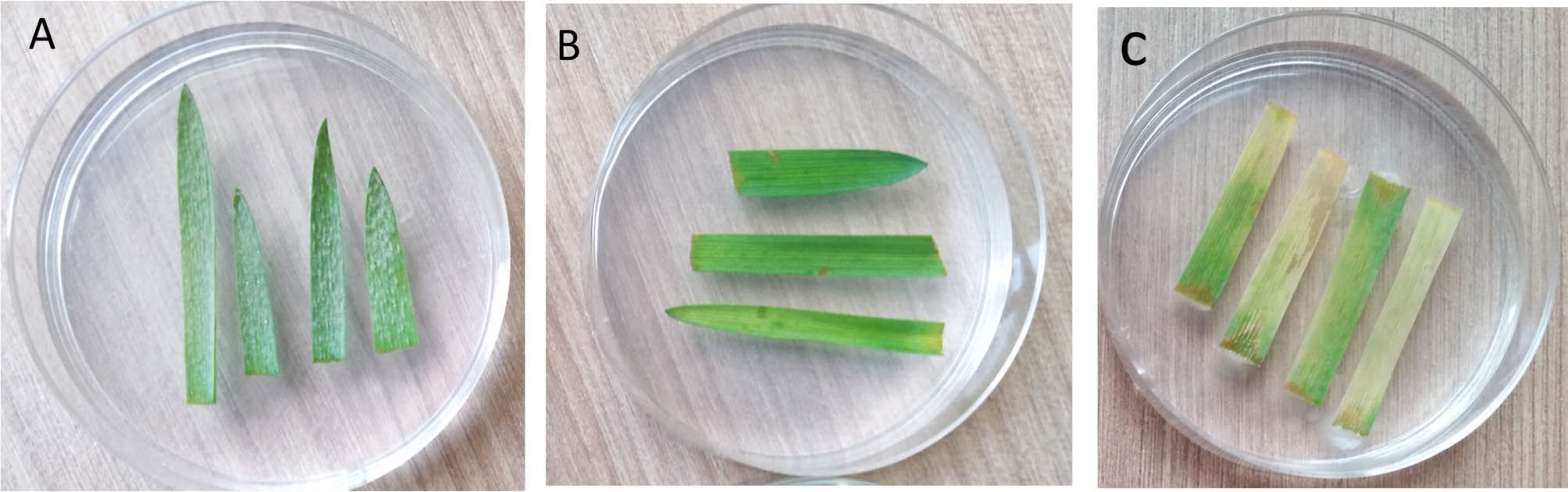
Example result of host-pathogen test conducted in vitro on leaf fragments in the presence of tested compounds. (**A**) control conditions - leaves of a susceptible oat variety (Fuchs) placed in a medium with DMSO additive inoculated with *B. graminis* f.sp. *avenae* spores. Clear symptoms of the disease are visible on the leaves in the form of a white, floury coating. (**B**) leaves of a susceptible oat variety (Fuchs) placed in a medium with 4 mg∙mL-1 coumarin derivative 6 inoculated with *B. graminis* f.sp. *avenae* spores. The leaves are green, with no visible symptoms of disease and no necrosis. (**C**) leaves of a susceptible oat variety (Fuchs) placed in a medium with 5 mg∙mL-1 coumarin derivative 6 inoculated with *B. graminis* f.sp. *avenae*. Numerous chloroses on leaves preventing proper assessment of the growth and development of the pathogen.

The analyzed compounds, which are coumarin derivatives, inhibited the growth and development of biotrophic fungal pathogens in diverse ways. The results of the host-pathogen tests are presented in Table 1. Initially, observations were conducted at a compound concentration of 5 mg∙mL^− 1^ of agar medium. The test yielded promising results; however, chlorosis and discoloration of entire leaves made it difficult to assess the degree of leaf infection by pathogens. To verify the effectiveness of the compounds, the test was repeated at a concentration of 4 mg∙mL^− 1^. An example test result illustrating the response of plants to the compounds used in the experiment is shown in Fig. 7 In this case, there were no issues with interpreting the results or evaluating the degree of leaf infection using the 5-point scale.

**Table 1:**
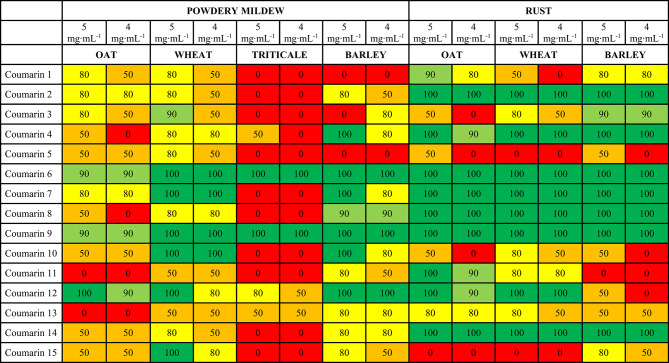
Results of the host-pathogen tests using a 5-point scale (1) 100% - complete inhibition of pathogen growth; (2) 90% - limited development of pathogens, single colonies; (3) 80% - visible mycelium with a small number of spores; (4) 50% - mycelium covering 20 to 50% of the leaf; (5) 0% mycelium covering more than 50% of the leaf; coumarin numbers refer to compounds in Figs. 2 and 3.

**Fig. 8:**
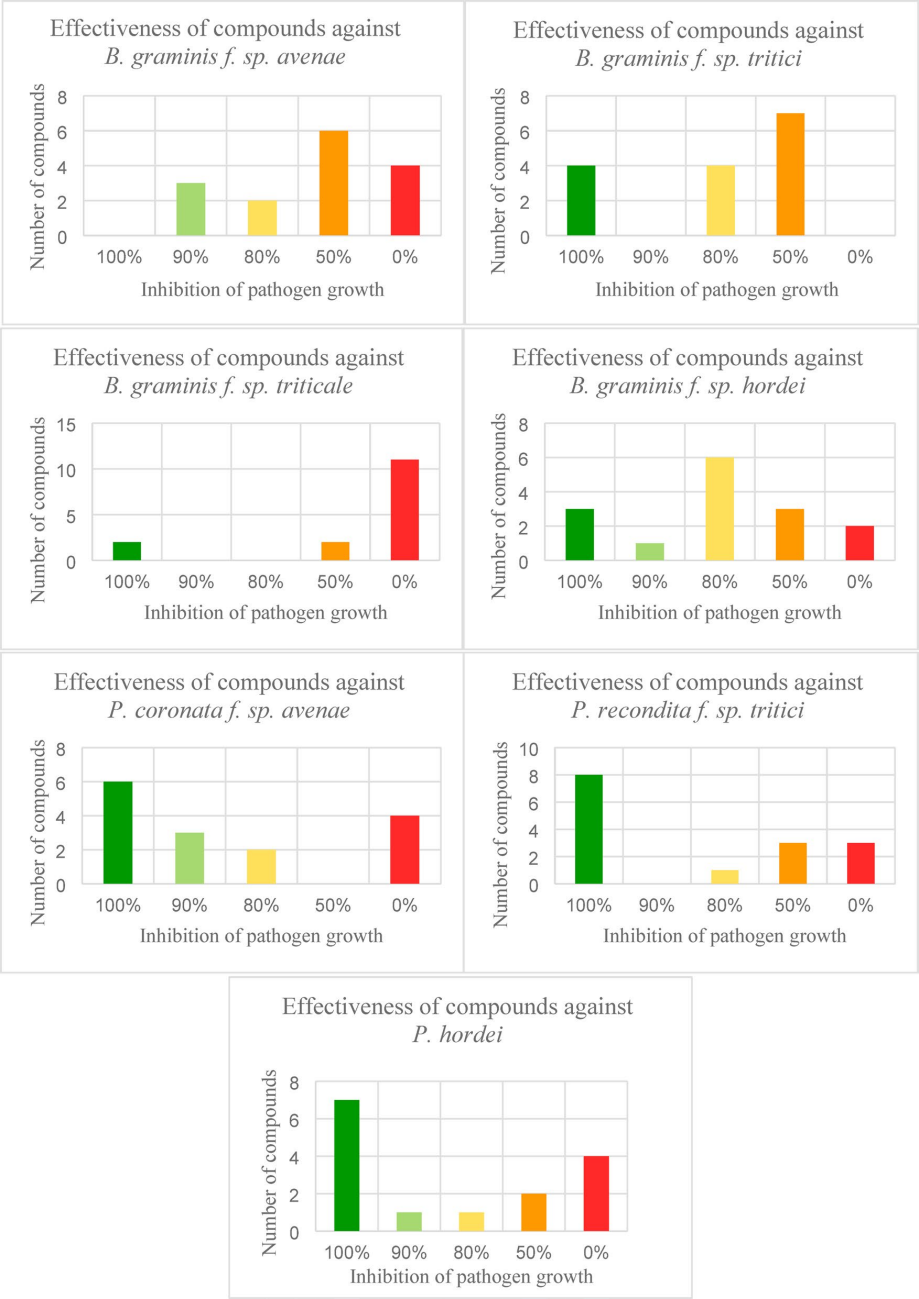
Effectiveness of the analyzed compounds in inhibiting the growth and development of fungal pathogens for the compound concentration of 4 mg∙mL^− 1^; number of analyzed compounds and their amount showing inhibition of the growth of fungal pathogens.

The detailed results for the compound concentration of 4 mg∙mL^− 1^ are presented in Fig. 8. Among the 15 analyzed compounds, none showed 100% ability to inhibit the growth of *B. graminis* f. sp. *avenae* actively. Three compounds exhibited 90% activity, while two inhibited development by 80%. Six compounds reduced fungal growth by 50%, and four compounds showed no effect on powdery mildew development. In the case of *B. graminis* f. sp. *tritici*, all 15 tested compounds demonstrated effectiveness ranging from 50% to 100%. Four compounds completely inhibited fungal cell growth, four showed 80% effectiveness, and the remaining seven inhibited development by 50%. Two compounds inhibited the growth and development of *B. graminis* f. sp. *Tritici* with 100% effectiveness, two others acted at a 50% level, and the remaining 11 compounds did not affect the development of this pathogen. Additionally, three out of the 15 compounds completely inhibited the disease caused by *B. graminis* f. sp. *hordei*. One compound inhibited fungal cell growth by 90%, six compounds showed 80% effectiveness, three inhibited fungal growth by 50%, and the remaining two had no effect on pathogen development.

Seven compounds inhibited the growth and development of all fungal pathogens of the genus *Blumeria* by 50–100%, while two compounds restricted the growth of these pathogens by 90–100%.

Against *P. coronata* f. sp. *avenae*, out of the 15 compounds, 6 showed 100% inhibition of fungal cell development, three compounds had 90% effectiveness, another two compounds acted with 80% effectiveness, while four compounds showed no noticeable effects. Another biotrophic fungus was *P. recondite* f. sp. *tritici*, whose development was inhibited by 100% by eight compounds. The effectiveness of 1 compound was estimated at 80%, three compounds inhibited cell growth by 50%, while no activity against the pathogen was observed for the remaining three compounds. On the other hand, seven compounds showed 100% activity in inhibiting *P. hordei*; one compound acted effectively at 90%, one inhibited fungal development at 80%, and two compounds reduced the incidence of the disease by 50%. In comparison, no changes in the normal development and growth of biotrophic fungi were observed for four compounds.

Six compounds inhibited the growth and development of all fungal pathogens of the genus *Puccinia* at a level of 100%, one compound restricted the growth of these pathogens at 90–100%, and one compound acted with an effectiveness of 50–80%.

The results of the conducted studies indicate that 2 out of the 15 tested compounds show effectiveness in inhibiting the growth and development of pathogens from both the *Blumeria* and *Puccinia* genera at a 90–100% level.

The introduction of various substituents into the basic coumarin compound has altered its biological properties. Depending on the functional groups, coumarin analogs exhibit varying degrees of ability to inhibit fungal pathogens. Compound 6, which turned out to be one of the two most effective in our study, in its structure—besides the benzene ring and the lactone derived from the basic coumarin—also contains an ester group and a Br substituent. The second compound, Compound 9, consists of a benzene ring condensed with a lactone, along with the attachment of an ester group and a Cl substituent. Compounds with such structures can acquire features responsible for diverse biological properties, including potential antifungal activity.

### Analysis of histological results

**Fig. 9:**
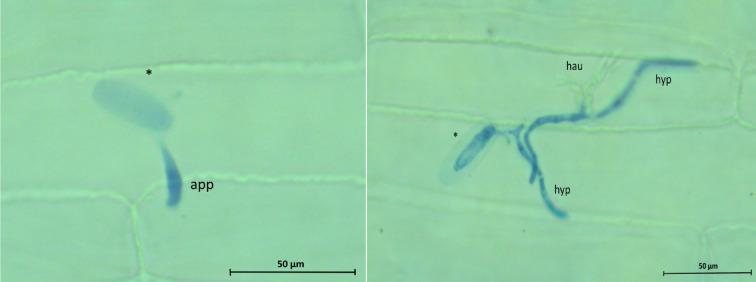
*Blumeria graminis* f. sp. *avena*e progression on oat leaves visualized with trypan blue at 48hpi. Black stars= conidium; app = appressorium; hau = haustoria, hyp = hyphae.

Microscopic analyses focused on the two most promising compounds (6 and 9) out of the 15 tested. A controlled trial involved fragments of leaves from susceptible cereals 48 h after inoculation with fungal pathogens, with DMSO as the additive. A research trial was also carried out, in which plant fragments enriched with selected coumarin derivatives were observed under a light microscope after 48 h of inoculation with the pathogens. In both the control and research trials, four developmental stages of the pathogen were observed (Fig. 9.): conidial spores; appressorium; haustorium; secondary infectious hyphae.

**Fig. 10:**
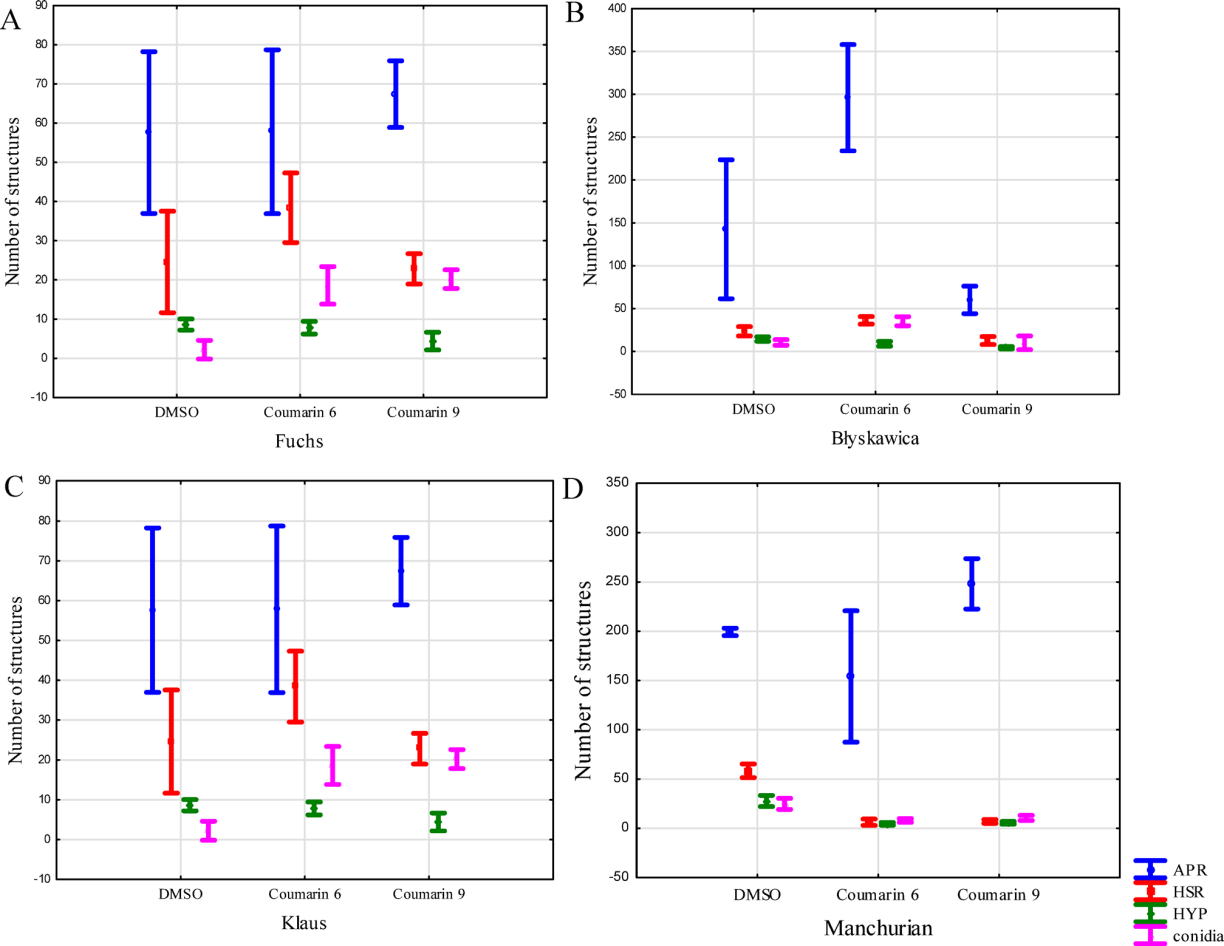
Statistical analyses of the occurrence of selected pathogen structures (*Blumeria graminis)* in plant cells: (**A**) – oats, (**B**) – wheat, (**C**) – triticale, (**D**) – barley; analyzed structures: APR - appressorium-stage spore with no penetration; HSR - successfully penetrated spore with haustorium, HYP - successfully-penetrated spore with developed hyphae penetrating into the intercellular spaces, conidia - ungerminated spores.

Statistical analyses of microscopic studies were conducted for control samples (without the addition of the tested compounds, with DMSO) and for two coumarin derivatives (**6**,** 9**) (Fig. 10.). The analysis focused on the comparison of the analyzed compounds (6,9) relative to the DMSO control. The remaining results of the Tukey test analysis are presented in supplementary materials (Figure S10-S25). The analyses focused on the occurrence of selected cellular structures in plant cells. In the case of *B. graminis* f. sp. *avenae*, compared to the control, coumarin 6 showed a significantly (*p* = 0.0332) higher level of haustoria occurrence. A significantly (*p* = 0.0018) lower occurrence of hyphae was observed for coumarin 9, while a significantly (*p* = 0.00019) higher occurrence of conidia was noted for both coumarin six and coumarin 9 (*p* = 0.00019). In other cases of fungal cell appearance, the differences between the control and the samples with the tested compounds were insignificant.

For *B. graminis* f. sp. *tritici*, it was observed that for coumarin 6, the occurrence of appressoria was significantly (*p* = 0.00091) higher, as was the occurrence of haustoria (*p* = 0.0008) and conidia (*p* = 0.00019). In comparison, the occurrence of hyphae was significantly (*p* = 0.00014) lower. In the case of coumarin 9, the occurrence of appressoria (*p* = 0.0462), haustoria (*p* = 0.00268), and hyphae (*p* = 0.00019) were significantly lower, while the occurrence of conidia showed no significant differences compared to the control sample.

For *B. graminis* f. sp. *tritici*, the occurrence of haustoria was significantly (*p* = 0.0332) higher for coumarin six than for the control sample, and the same was true for conidia (*p* = 0.00019). In other cases, coumarin 6 showed no significant differences. The occurrence of appressoria and haustoria in the sample with coumarin nine also showed no significant differences, while the presence of hyphae was significantly (*p* = 0.00182) lower, and conidia were significantly (*p* = 0.00019) higher.

The final statistical analysis included the occurrence of sporulation structures of the pathogen *B. graminis* f. sp. *hordei*. The presence of appressoria showed no significant differences between the tested compounds and the control. However, in the samples with coumarin 6, a significantly lower level of occurrence was observed for haustoria (*p* = 0.00019), hyphae (*p* = 0.00019), and conidia (*p* = 0.00019). For coumarin 9, the occurrence of haustoria (*p* = 0.00019), hyphae (*p* = 0.00019), and conidia (*p* = 0.0002) was also significantly lower.

The data obtained from the statistical analyses suggest that the development of individual fungal pathogens proceeded normally in both the control and treated samples, with the addition of coumarin 6 and coumarin 9. However, the occurrence level of sporulation structures changed, as evidenced by the Tukey test analysis and the presentation of the results in a box plot.

##  Discussion

There are several ways to protect cereals against harmful fungal pathogens. These include agrochemical, biological, genetic, and chemical methods. Agrotechnical methods involve introducing crop rotation into cultivation, as well as fertilization, for example with manure, and selecting the appropriate sowing time and density. On the other hand, biological methods involve the use of appropriate biological preparations that utilize other microorganisms; plant resistance stimulants are also used as factors supporting the defense mechanisms of cereals. The genetic method refers to a procedure in which cereal varieties resistant or tolerant to fungal pathogens are used in cultivation. The last chemical method of combating pathogens is the use of fungicides or seed treatment to prevent disease transmission^42^. These strategies are often used in combination as part of integrated plant protection programs, which aim to balance efficacy with environmental sustainability^43^.

In agriculture, there is an ongoing search for plant protection products that are both effective and environmentally friendly, which is particularly important in cereal crops. Fungal pathogens are developing increasing resistance to traditionally used plant protection products, rendering them ineffective against diseases. Additionally, in line with the EU’s Farm to Fork (F2F) Strategy and the EU Biodiversity Strategy for 2030, the number of available chemical plant protection products will be reduced by 50% by 2030. Therefore, researchers focus on discovering and developing new, effective fungicides^44^. Many plant protection products available on the market are based on natural compounds. This has prompted researchers to experiment with coumarin, a naturally occurring environmental substance, and its derivatives. Interestingly, numerous coumarin analogues, once extracted, are widely used in agrochemistry. It has been discovered that modifying the structure of these compounds enhances their biological activities and imparts new properties^30^. Such functionality has made this group of compounds widely studied not only for their application in agriculture as insecticides^45^, acaricides^46^, bactericides^47^, and fungicides^48^ but also in medicine and pharmacology as sedatives, anticancer agents, antioxidants, and anti-inflammatory agents^27^.

Our results contribute to the extensive literature on the antifungal activity of coumarin compounds. These studies emphasize that coumarins are natural phytoalexins induced by plants in response to infection and inhibit the growth of pathogens^49^.One of the commercially used coumarin-based fungicides in agriculture is Osthole. This product is used to combat powdery mildew in wheat, powdery mildew in cucumbers, and downy mildew in grapes. This fungicide induces autophagy, a process that destroys the cellular structure of the fungus^31^. Another commonly used plant protection product containing coumarin is coumoxystrobin. It has a broad spectrum of activity against fungal pathogens causing powdery mildew, downy mildew, grey mould, and sheath blight^50^. However, prolonged use of a specific fungicide can lead to plant resistance, ultimately rendering it ineffective. This situation underscores the importance of developing new products that may serve as plant protection agents in the future^51^.

In searching for biologically active agents that could serve as fungicides, scientists have researched natural compounds from the coumarin group. These studies involved modifying the structure of the compounds by attaching selected functional groups to the basic framework, thereby creating analogues^52^. Literature data report numerous coumarin compounds that effectively combat fungal plant diseases^30^. It should be noted that most studies, including our own research, were conducted in vitro, which poses significant limitations when extrapolating results to natural infections. Biotrophic cereal pathogens require living tissue and do not reproduce on artificial media^53^, meaning our experimental model was simplified. Furthermore, dimethyl sulfoxide (DMSO) was used to dissolve the tested compounds. Although the DMSO concentration used was low and the negative control did not demonstrate significant inhibition of fungal growth, its subtle effects on the pathogen or plant cannot be completely ruled out. It is also worth noting that at higher concentrations of the tested substances, symptoms of phytotoxicity – chlorosis of host leaves – were observed. As in other studies, it was noted that even at moderate doses, the coumarin compound can inhibit plant growth and cause a reduction in photosynthetic pigment content^34^. These observations emphasize the need to carefully adjust the dosage and formulation of compounds and use appropriate controls to separate antipathogenic effects from potential side effects on the plant. However, such studies allow for the exploration of the potential of new compounds and indicate which ones should be the subject of more detailed investigation.

Our research included an assessment of the impact of selected compounds on the growth and development of fungal pathogens. Each of these compounds contained two fused rings: a benzene ring and a pyran ring, along with substituted selected functional groups. The selection of appropriate substituents influences the alteration of antifungal activity in compounds. Coumarin analogs, which almost completely inhibited the growth and development of fungal pathogens, proved to be much more effective than coumarin itself. As it turns out, the highest efficacy was exhibited by compounds whose structure consisted of a benzene ring and a lactone derived from the basic coumarin, as well as an ester group and a Br or Cl substituent. Additionally, high inhibition of the growth and development of biotrophic fungi was confirmed by control sample analyses lacking the tested compounds and containing only the solvent in which the derivatives were dissolved—DMSO. In the control sample, the mycelium covered almost the entire leaf structure, whereas the effective coumarin analogs with new biological properties significantly limited pathogen development^54^. While the antifungal effects observed were substantial, it is important to interpret these findings cautiously, as compound efficacy may vary across pathogen species and plant genotypes in field conditions. These coumarin derivatives exhibited enhanced or entirely new biological activities. The tested agents exhibiting the most substantial antifungal properties showed complete inhibition of fungal cell development, ranging from 90 to 100%. These results confirmed that the studied compounds could potentially be applied in the future as plant protection agents demonstrating vigorous biological activity against biotrophic fungal pathogens. This indicates the high potential of the tested agents, which could be utilized in agriculture^55^.

On the other hand, microscopy revealed that the studied cereal species exhibited resistance to fungal pathogen attacks when exposed to the selected compounds. For the chosen coumarin derivatives (**6**,** 9**), the studies initially confirmed the normal development of pathogens - conidial spores germinated, initiating host infection. The appressorium formed at the end of the germ tube allowed the conidial spores to attach to the infected plant. The next stage of infection involved the growth of the infection hypha and the emergence of haustoria penetrating plant cells. However, as indicated by the experiment conducted on the studied cereals with the addition of the compounds compared to the control sample, there was a reduced number of haustoria. This results from the plant forming papillae as a response to the pathogen attack. A small number of haustoria continue to penetrate the cells and develop into hyphae. However, their limited quantity may indicate that the plant produced an encasement around the haustoria aimed at blocking access to nutrients extracted from the host cell^56^. However, since only selected stages of infection were microscopically evaluated, it remains unclear whether later systemic responses or long-term resistance mechanisms were also affected by the compounds.

## Conclusions

In summary, the results of our research demonstrated that selected coumarin derivatives exhibited diverse inhibitory effects on the development of biotrophic fungal pathogens. Host plant tests revealed that at a concentration of 4 mg∙mL^− 1^, the majority of the 15 tested compounds effectively inhibited fungal growth, with four compounds showing 100% efficacy in suppressing pathogen growth. Coumarin derivatives 6 and 9 stood out for their particularly high effectiveness, which was confirmed by microscopic analysis. Histological analysis showed that these compounds reduced the occurrence of pathogen structures such as haustoria, appressoria, hyphae, and conidia, suggesting their potential in modulating plant-pathogen interactions. Further microbiological studies indicated significant differences in the occurrence of sporulation structures in samples treated with coumarin derivatives, which may point to their potential in limiting pathogen spread.

Coumarin 6 and 9 demonstrated a clear reduction in the presence of mycelium and haustoria, which is a promising result for the development of new plant protection agents. It is worth emphasizing that in tests on various plant species, such as oats, wheat, barley, and rye, these compounds showed varying efficacy depending on the fungal species. Although none of the tested compounds achieved 100% efficacy in all tests, the results suggest great promise for these coumarin derivatives as future plant protection agents in agriculture. However, further research is necessary to optimize their use and understand their mechanisms of action in order to fully harness their potential in protecting against fungal diseases.

## Materials and methods

### Chemistry

Commercially available chemicals were obtained from Sigma-Aldrich and used as received. NMR spectra were recorded using a Bruker AV500 (^1^H 500 MHz, ^13^C NMR 126,) spectrometer. All spectra were obtained in CDCl_3_ solutions, and the chemical shifts (δ) were expressed in ppm using internal reference to TMS. Coupling constants (*J*) were given in Hz. The abbreviations of signal patterns were as follows: s, singlet; d, doublet; t, triplet; q, quartet; m, multiplet; b, broad. Thin-layer chromatography (TLC) was done on silica gel (Kieselgel 60, F254 on aluminum sheets, Merck) using UV light (254 nm). All column chromatographic separations and purifications were conducted using Merck silica gel 60 (230–400 mesh). HPLC–HRMS was performed on a Shimazu LCMS-8030 LCMS System using a reverse-phase stationary phase with water/MeCN (65:35) as an eluent, electrospray ionization (ESI), and an IT-TOF detector (Shimadzu Europa, Duisburg, Germany).

#### General procedure for synthesis of coumarin-3-carboxylic acid allyl esters (1–6)

Salicylaldehyde (6.0 g, 0.05 mol), diallyl malonate (1.05 eq.), *L*-proline (0.2 eq.), and acetonitrile (25 ml) were placed in a Schlenk tube. The mixture was stirred at 80 °C for 18 h. Subsequently, the volume of the solvent was halved through evaporation, and the pure coumarin was obtained by crystallization from ethanol at 4 °C.

*Allyl 2-oxo-2 H-chromene-3-carboxylate (* ***1*** *).*

White solid, M.p. 50–52 °C, yield 85%, ^1^H NMR (500 MHz) δ 8.53 (s, 1 H), 7.64–7.60 (m, 2 H), 7.34–7.30 (m, 2 H), 6.00 (tdd, *J* = 16.4, 5.6, 1.9 Hz, 1 H), 5.46 (d, *J* = 16.0 Hz, 1H), 5.45 (d, *J* = 17.0 Hz, 1H), 4.84 (d, *J* = 5.6 Hz, 2 H). ^13^C NMR (126 MHz) δ 162.6, 156.6, 155.1, 148.9, 134.4, 131.5, 129.5, 124.8, 119.0, 117.7, 116.7, 66.3. The NMR data are consistent with the values reported in the literature^57^.

*Allyl 7-methoxy-2-oxo-2 H-chromene-3-carboxylate (****2****)*.

White solid, M.p. 100–103 °C, yield 70%, ^1^H NMR (500 MHz) δ 8.56 (s, 1H), 7.53 (d, *J* = 8.8 Hz, 1H), 6.92 (dd, *J* = 8.5, 2.5 Hz, 1H), 6.85 (d, *J* = 2.2 Hz, 1H), 6.09–6.02 (m, 1H), 5.49 (ddd, *J* = 17.0, 2.8, 1.6 Hz, 1H), 5.33 (ddd, *J* = 10.4, 2.5, 1.2 Hz 1H), 4.86 (dt, *J* = 5.7, 1.6 Hz, 2 H), 3.93 (s, 3 H). ^13^C NMR (126 MHz) δ 165.2, 163.1, 157.6, 157.0, 149.3, 131.7, 130.8, 118.9, 113.7, 111.6, 100.4, 66.2, 56.0. The NMR data are consistent with the values reported in the literature^57^.

*Allyl 8-methoxy-2-oxo-2 H-chromene-3-carboxylate (****3****)*.

White solid, M.p. 95–197 °C, yield 90%, ^1^H NMR (500 MHz) δ 8.52 (s, 1H), 7.28–7.24 (m, 1H), 7.19–7.17 (m, 2 H), 6.03–5.99 (m, 1H), 5.55 (ddd, *J* = 17.2, 2.8, 1.6 Hz, 1H), 5.32 (ddd, *J* = 10.4, 2.5, 1.2 Hz, 1H), 4.84 (dt, *J* = 5.7, 1.6 Hz, 2 H), 3.97 (s, 3 H). ^13^C NMR (126 MHz) δ 162.7, 156.0, 149.1, 147.0, 144.8, 131.5, 124.8, 120.6, 119.0, 118.4, 118.1, 116.0, 100.4, 66.4, 56.3. The NMR data are consistent with the values reported in the literature^57^.

*Allyl 6-bromo-2-oxo-2 H-chromene-3-carboxylate (****4****)*.

White solid, M.p. 154–155 °C, yield 72%, ^1^H NMR (500 MHz) δ 8.48 (s, 1H), 7.78–7.74 (m, 2 H), 7.28 (d, *J* = 8.8 Hz, 1H), 6.09–6.01 (m, 1H), 5.51–5.45 (m, 1H), 5.36–5.34 (m, 1H), 4.88–4.86 (m, 1H). ^13^C NMR (126 MHz) δ 163.2, 155.9, 154.0, 147.4, 137.1, 131.6, 131.3, 119.3, 118.6, 117.4, 66.56. The NMR data are consistent with the values reported in the literature^58^.

*Allyl 6*,*8-dibromo-2-oxo-2 H-chromene-3-carboxylate (****5****)*.

Brown solid, M.p. 152–155 °C, yield 54%, ^1^H NMR (500 MHz) δ 8.43 (s, 1H), 8.01 (d, *J* = 2.21 Hz, 1H), 7.72 (d, *J* = 2.2 Hz, 1H), 6.07-6.00 (m, 1H), 5.50 (ddd, *J* = 17.3, 3.1, 1.6 Hz, 1H), 5.36 (ddd, *J* = 10.4, 2.5, 1.2 Hz, 1H), 4.87 (dt, *J* = 5.7, 1.5 Hz, 2 H). ^13^C NMR (126 MHz) δ 162.5, 162.0, 157.9, 154.8, 147.0, 139.2, 131.2, 130.8, 120.0, 119.3, 117.3, 66.8. HRMS (ESI-IT-TOF) m/z calcd for C_13_H_8_Br_2_O_4_ [M + H]+: 386.8789; found: 386.8790.

*Allyl 3-oxo-3 H-benzo[f]chromene-2-carboxylate (****6****)*.

White solid, M.p. 113–115 °C, yield 54%, ^1^H NMR (500 MHz) δ 9.34 (s, 1H), 8.30 (d, *J* = 8.2 Hz, 1H), 8.11 (d, *J* = 9.1 Hz, 1H), 7.94 (d, *J* = 8.2 Hz, 1H), 7.77 (t, *J* = 8.2 Hz, 1H), 7.63 (t, *J* = 7.0 Hz, 1H), 7.45 (d, *J* = 8.8 Hz, 1H), 6.13–6.08 (m, 1H), 5.54 (ddd, *J* = 17.2, 2.8, 1.6 Hz, 1H), 5.38 (ddd, *J* = 10.4, 2.5, 1.3 Hz, 1H), 4.93 (dt, *J* = 5.6, 1.6 Hz, 2 H). ^13^C NMR (126 MHz) δ 163.2, 156.7, 156.1, 144.8, 136.3, 131.6, 130.2, 129.3, 129.2, 126.6, 121.4, 119.1, 116.7, 116.1, 112.2, 66.5. The NMR data are consistent with the values reported in the literature^57^.

*Allyl 6*,*8-dibromo-2-oxo-2 H-chromene-3-carboxylate (****7****)*.

Brown solid, M.p. 152–155 °C, yield 54%, ^1^H NMR (500 MHz) δ 8.43 (s, 1H), 8.01 (d, *J* = 2.21 Hz, 1H), 7.72 (d, *J* = 2.2 Hz, 1H), 6.07-6.00 (m, 1H), 5.50 (ddd, *J* = 17.3, 3.1, 1.6 Hz, 1H), 5.36 (ddd, *J* = 10.4, 2.5, 1.2 Hz, 1H), 4.87 (dt, *J* = 5.7, 1.5 Hz, 2 H). ^13^C NMR (126 MHz) δ 162.5, 162.0, 157.9, 154.8, 147.0, 139.2, 131.2, 130.8, 120.0, 119.3, 117.3, 66.8. The NMR data are consistent with the values reported in the literature^57^.

*Allyl 3-oxo-3 H-benzo[f]chromene-2-carboxylate (****8****)*.

White solid, M.p. 113–115 °C, yield 54%, ^1^H NMR (500 MHz) δ 9.34 (s, 1H), 8.30 (d, *J* = 8.2 Hz, 1H), 8.11 (d, *J* = 9.1 Hz, 1H), 7.94 (d, *J* = 8.2 Hz, 1H), 7.77 (t, *J* = 8.2 Hz, 1H), 7.63 (t, *J* = 7.0 Hz, 1H), 7.45 (d, *J* = 8.8 Hz, 1H), 6.13–6.08 (m, 1H), 5.54 (ddd, *J* = 17.2, 2.8, 1.6 Hz, 1H), 5.38 (ddd, *J* = 10.4, 2.5, 1.3 Hz, 1H), 4.93 (dt, *J* = 5.6, 1.6 Hz, 2 H). ^13^C NMR (126 MHz) δ 163.2, 156.7, 156.1, 144.8, 136.3, 131.6, 130.2, 129.3, 129.2, 126.6, 121.4, 119.1, 116.7, 116.1, 112.2, 66.5. The NMR data are consistent with the values reported in the literature^57^.

#### General procedure for the esterification reaction with Oxone

Coumarin-3-carboxylic acid (3.0 g, 0.015 mol), Oxone (0.5 eq., 2.42 g), alcohol (3.0 eq., 0.045 mol), and acetonitrile (25 ml) were placed in a Schlenk tube. The mixture was stirred at 80 °C for 48 h. The reaction mixture was subsequently diluted with ethyl acetate and washed with a 5% sodium bicarbonate solution and water. The organic layer was dried using anhydrous Na_2_SO_4_, subsequently filtered, and concentrated under reduced pressure. Excess alcohol was distilled under reduced pressure. The product was purified using column chromatography, eluting with ethyl acetate/hexane (1/10).

*3-Chloropropyl 2-oxo-2 H-chromene-3-carboxylate (****9****)* White solid, M.p. 90–93 °C, yield 72%, ^1^H NMR (500 MHz, CDCl_3_) δ 8.57 (s, 1H), 7.74–7.62 (m, 2 H), 7.44–7.35 (m, 2 H), 4.54 (t, *J* = 6.0 Hz, 2 H), 3.78 (t, *J* = 6.3 Hz, 2 H), 2.32–2.24 (m, 2 H). ^13^C NMR (126 MHz) δ 13 C NMR (126 MHz, CDCl3) δ 163.2, 156.6, 155.2, 149.0, 134.5, 129.6, 124.9, 118.0, 117.8, 116.8, 62.5, 41.2, 31.4.

*2-Hydroxyethyl 2-oxo-2 H-chromene-3-carboxylate (* ***10*** *)* White solid, M.p. 130–133 °C, yield 81%, ^1^H NMR (500 MHz, CDCl_3_) δ 8.59 (s, 1H), 7.72–7.64 (m, 2 H), 7.44–7.37 (m, 2 H), 4.54–4.48 (m, 2 H), 3.98 (s, 2 H). ^13^C NMR (126 MHz, CDCl_3_) δ 163.61, 155.19, 149.22, 134.61, 129.63, 125.03, 118.16, 117.83, 116.88, 67.47, 60.81. The NMR data are consistent with the values reported in the literature^59^.

#### General procedure for the Steglich esterification reaction

A solution of coumarin-3-carboxylic acid (**2**, 1.0 g, 5.26 mmol) in dry DCM (15 mL) was treated with DCC (1.19 g, 5.78 mmol), DMAP (0.032 g, 0.26 mmol), and the corresponding alcohol (5.78 mmol). The reaction mixture was stirred at room temperature for 48 h. The subsequent by-product DCU (*N*,* N’*-dicyclohexylurea) precipitate was removed from the reaction mixture via filtration, and the filtrate was further concentrated. The produced coumarin esters were purified using column chromatography (hexane/ethyl acetate 15:1) and/or recrystallisation employing ethanol.

*Cinnamyl 2-oxo-2 H-chromene-3-carboxylate (* ***11*** *)* White solid, M.p. 114–116 °C, yield 82%, ^1^H NMR (500 MHz, CDCl_3_) δ 8.61 (s, 1H), 7.73–7.63 (m, 2 H), 7.53–7.30 (m, 7 H), 6.82 (d, *J* = 15.9 Hz, 1H), 6.49–6.41 (m, 1H), 5.05 (dd, *J* = 6.5, 1.3 Hz, 2 H). ^13^C NMR (126 MHz, CDCl_3_) δ 162.86, 156.66, 155.25, 149.00, 136.07, 135.14, 134.49, 129.56, 128.64, 128.23, 126.74, 124.88, 122.43, 118.05, 117.87, 116.86, 66.47. The NMR data are consistent with the values reported in the literature^60^.

*(1R*,*2 S*,*5R)-2-isopropyl-5-methylcyclohexyl 2-oxo-2 H-chromene-3-carboxylate (* ***12*** *).* White solid, M.p. 144–146 °C, yield 65%, [α]^D^_20_ -58.5 (c 0.55, DCM) ^1^H NMR (500 MHz): δ 8.49 (s, 1H), 7.69–7.62 (m, 2 H), 7.41–7.33 (m, 2 H), 4.99 (td, *J* = 10.9, 4.4 Hz, 1H), 2.20–1.98 (m, 2 H), 1.81–1.53 (m, 6 H), 1.23–1.07 (m, 1H), 0.97 (d, *J* = 3.4 Hz, 3 H), 0.95 (d, *J* = 3.8 Hz, 3 H), 0.83 (d, *J* = 6.9 Hz, 3 H). ^13^C NMR (126 MHz): δ 162.5, 156.6, 155.1, 147.9, 134.1, 129.4, 124.7, 118.7, 117.9, 116.7, 76.0, 46.9, 40.7, 34.1, 31.4, 26.1, 23.3, 22.0, 20.8, 16.2. The NMR data are consistent with the values reported in the literature^61^. CCDC No. 2,329,677.

3,7-Dimethyloct-6-en-1-yl 2-oxo-2 H-chromene-3-carboxylate (**13**)

Colourless oil, yield 82%, ^1^H NMR (500 MHz): δ 8.53 (s, 1H), 7.72–7.59 (m, 2 H), 7.37 (ddd, *J* = 9.0, 8.4, 4.7 Hz, 2 H), 5.18–5.08 (m, 1H), 4.47–4.35 (m, 2 H), 2.03 (qd, *J* = 14.4, 7.3 Hz, 2 H), 1.90–1.82 (m, *J* = 7.9, 2.7 Hz, 2 H), 1.70 (s, 3 H), 1.67–1.55 (m, 5 H), 1.46–1.36 (m, 1H), 1.38–1.21 (m, 1H), 0.99 (d, *J* = 5.0 Hz, 3 H). ^13^C NMR (126 MHz): δ 163.0, 156.6, 155.1, 148.4, 134.3, 131.3, 129.5, 124.8, 124.5, 118.3, 117.8, 116.7, 64.5, 36.9, 35.3, 29.5, 25.7, 25.3, 19.4, 17.6. HRMS (ESI-IT-TOF) m/z calcd for C_20_H_24_O_4_ [M + H]+: 329.1675; found: 329.1677.

*(Diphenylphosphoryl)methyl 2-oxo-2 H-chromene-3-carboxylate (****14****)* White solid, M.p. 123–126 °C, yield 89%, ^1^H NMR (500 MHz, CDCl_3_) δ 8.43 (s, 1H), 8.00–7.92 (m, 4 H), 7.71–7.51 (m, 8 H), 7.40–7.34 (m, 2 H), 5.11 (d, *J* = 5.6 Hz, 2 H). ^13^C NMR (126 MHz, CDCl_3_) δ 162.11 (d, *J* = 7.8 Hz), 162.07, 156.29, 155.28, 150.14, 134.98, 132.73, 131.54 (d, *J* = 9.8 Hz), 129.87, 129.30, 128.91, 128.86 (d, *J* = 12.2 Hz), 125.05, 117.65, 116.81, 116.56. ^31^P (202 MHz) δ 26.52. HRMS (ESI-IT-TOF) m/z calcd for C_23_H_18_O_5_P+ [M + H]+: 405.0814; found: 405.0816.

#### Synthesis of 4-(diphenylphosphoryl)chroman-2-one *(****15****)*

To a solution of coumarin-3-carboxylic acid (**2**, 1.0 g, 5.26 mmol) in water (25 mL) diphenylphosphine oxide (1.06 g, 5.26 mmol) was added and the reactions were stirred vigorously. After a time of 4 h, the precipitated product was filtered off and dried under vacuum.

White solid, M.p. 265–268 °C, yield 92%, ^1^H NMR (500 MHz, CDCl_3_) δ 8.05–7.37 (m, 11 H), 7.19–6.57 (m, 3 H), 4.29–3.94 (m, 1H), 3.47–3.32 (m, 1H), 2.87 (ddd, *J* = 26.3, 13.1, 7.4 Hz, 1H). ^13^C NMR (126 MHz, DMSO-*d*_6_) δ 166.08, 152.53, 132.28 (d, *J* = 14.2 Hz), 131.92, 131.28, 130.96, 130.92, 130.65, 128.83, 128.35 (d, *J* = 3.8 Hz), 129.15, 128.90, 128.54, 123.49, 117.99, 116.82, 42.68, 35.25, 31.25. ^31^P (202 MHz) δ 33.04. The NMR data are consistent with the values reported in the literature^41^.

### Biological activity

The biological activity of the studied coumarin derivatives was analyzed under in vitro conditions using physiological host-pathogen tests. A total of 100 µg of the compounds were weighed and dissolved in 1 mL of DMSO. The agar medium was prepared by dissolving 6 g of agar in 1 L of water. Then, 50 µL of the compound was added to 10 mL of the agar medium to achieve a concentration of 5 µg∙mL^− 1^. Due to the occurrence of severe chlorosis on cereal leaves, a concentration of 4 µg∙mL^− 1^ was also tested in the subsequent part of the experiment. Leaves of susceptible cereal varieties were placed on Petri dishes containing agar medium supplemented with coumarin derivatives. Each dish was covered with 3 leaves from 3 different plants of a given species. A control test was performed on leaves placed on agar medium with the addition of pure DMSO. The cereal varieties and pathogens used in the experiment are presented in Table 2. Leaf fragments were inoculated with fungal pathogen spores by spraying the spores in inoculation towers. The plates with leaf fragments were then incubated at a temperature of 17 °C and a light intensity of 4 kLx. After 10 days of incubation, the degree of infection of the leaf fragments was assessed using a 5-point scale (Table 3). To confirm the correct result, the tests were performed in three independent repetitions. The percentage results refer to the control sample, where 100% indicates complete inhibition of fungal pathogen development, while 0% indicates no disease control ^11,^^55,56^.

**Table 2:** Cereal varieties and pathogens causing powdery mildew and rusts. The grains of the tested varieties were kindly provided by: Małopolska Breeding Company - Błyskawica (wheat), Saaten Union - Klaus (Triticale), Prof. Sai L.K. Hsam from the Technical University of Munich, Germany - Fuchs (oats), Prof. Paweł Czembor (IHAR BiP, Poland) - Manchurian (barley). Pathogen isolates came from the collection of the Institute of Genetics, Breeding and Biotechnology of Plants, University of Life Sciences in Lublin.

Cereal cultivar	Pathogen	Disease
Oat Fuchs	*Blumeria graminis f.sp. avenae*,	Powdery mildew
	*Puccinia coronata f.sp. avenae*	Crown rust
Wheat Błyskawica	*Blumeria graminis f.sp. tritici*,	Powdery mildew
	*Puccinia recondita f.sp.tritici.*	Brown rust
Barley Manchurian,	*Puccinia hordei*	Leaf rust
	*Blumeria graminis f.sp. hordei*,	Powdery mildew
Triticale Klaus	*Blumeria graminis f. sp. triticale*	Powdery mildew

**Table 3:** A 5-point scale for assessing oat leaf infection by fungal pathogens.

The degree of infection	Description of the degree	Percentage disease control
0	Lack of symptoms	100%
1	Limited development of the pathogen visible as single and small colonies	90%
2	Mycelium visible with a small quantity of spores - less than 20% of the leaf area	80%
3	Extensive mycelium occupying 20% − 50% leaf area	50%
4	Abundant mycelium occupying more than 50% of the leaf surface	0%

### Histological analysis of seedling resistance

Fragments (2–3 cm) of infected leaves were collected 48 h post inoculation (hpi) and boiled in trypan blue solution for 20 min^62^. Leaf fragments were then placed in chloral hydrate (2.5 g∙mL^− 1^ H_2_O) overnight. Fragments were rinsed in water and mounted on microscope slides with 80% glycerol. Slides were examined using a Leica DM5500B microscope under bright field conditions to determine the stage of infection of *Blumeria graminis* conidiospores. Histological analyses were performed for all cereal species infected with spores. The experiment was performed in three independent replicates, reading a minimum of 50 spores per leaf fragment. For each form, 6 fragments were analyzed, resulting in readings from 300 spores for each species. Percentage data were calculated from microscopic counts and fitted to a generalized linear model with a quasi-binomial distribution. Tukey’s HSD was performed to compare differences between the frequency of each resistance response expressed in each oat accession^63^.

## Supplementary Information

Below is the link to the electronic supplementary material.

Supplementary Material 1

## Data Availability

The datasets used and analysed during the current study are available from the corresponding author on reasonable request.

## References

[1] Waksman, S. A. & Associative and antagonistic effects of microorganisms: I. historical review of antagonistic relationships. *Soil Sci.* **43**, 51 (1937).

[2] Newton, A. C., Fitt, B. D. L., Atkins, S. D., Walters, D. R. & Daniell, T. J. Pathogenesis, parasitism and mutualism in the trophic space of microbe–plant interactions. *Trends Microbiol.* **18**, 365–373 (2010). 20598545 10.1016/j.tim.2010.06.002

[3] Casadevall, A. & Pirofski, L. The damage-response framework of microbial pathogenesis. *Nat. Rev. Microbiol.* **1**, 17–24 (2003). 15040176 10.1038/nrmicro732 PMC7097162

[4] Huang, Y. J. et al. Quantitative resistance to symptomless growth of Leptosphaeria maculans (phoma stem canker) in Brassica napus (oilseed rape). *Plant. Pathol.* **58**, 314–323 (2009).

[5] Glazebrook, J. Contrasting mechanisms of defense against biotrophic and necrotrophic pathogens. *Annu. Rev. Phytopathol.* **43**, 205–227 (2005). 16078883 10.1146/annurev.phyto.43.040204.135923

[6] Dean, R. et al. The Top 10 fungal pathogens in molecular plant pathology. *Mol. Plant. Pathol.* **13**, 414–430 (2012). 22471698 10.1111/j.1364-3703.2011.00783.x PMC6638784

[7] Fei, W. & Liu, Y. Biotrophic fungal pathogens: a critical overview. *Appl. Biochem. Biotechnol.* **195**, 1–16 (2023). 35951248 10.1007/s12010-022-04087-0

[8] Mendgen, K. & Hahn, M. Plant infection and the establishment of fungal biotrophy. *Trends Plant Sci.* **7**, 352–356 (2002). 12167330 10.1016/s1360-1385(02)02297-5

[9] O’Connell, R. J. et al. Dissecting the cell biology of Colletotrichum infection processes. in (eds Freeman, S., Dickman, M. & Prusky, D.) 55–77 (American Phytopathological Society (APS), (2000).

[10] Lorrain, C., Gonçalves Dos Santos, K. C., Germain, H., Hecker, A. & Duplessis, S. Advances in understanding obligate biotrophy in rust fungi. *New. Phytol*. **222**, 1190–1206 (2019). 30554421 10.1111/nph.15641

[11] Rząd, K. et al. Investigation of 2,4-dihydroxylaryl-substituted heterocycles as inhibitors of the growth and development of biotrophic fungal pathogens associated with the most common cereal diseases. *Int. J. Mol. Sci.* **25**, 8262 (2024). 39125838 10.3390/ijms25158262 PMC11312687

[12] Zhang, Z. et al. Of genes and genomes, needles and haystacks: Blumeria graminis and functionality. *Mol. Plant Pathol.* **6**, 561–575 (2005). 20565680 10.1111/j.1364-3703.2005.00303.x

[13] Both, M., Csukai, M., Stumpf, M. P. H. & Spanu, P. D. Gene expression profiles of Blumeria graminis indicate dynamic changes to primary metabolism during development of an obligate biotrophic pathogen. *Plant. Cell.* **17**, 2107–2122 (2005). 15951491 10.1105/tpc.105.032631 PMC1167555

[14] Carver, T. L. W. & Griffiths, E. Relationship between powdery mildew infection, green leaf area and grain yield of barley. *Ann. Appl. Biol.* **99**, 255–266 (1981).

[15] Roderick, H. W., Jones, E. R. L. & Šebesta, J. Resistance to oat powdery mildew in Britain and Europe: a review. *Ann. Appl. Biol.* **136**, 85–91 (2000).

[16] McTaggart, A. R. et al. Host jumps shaped the diversity of extant rust fungi (Pucciniales). *New Phytol.* **209**, 1149–1158 (2016). 26459939 10.1111/nph.13686

[17] Tavares, S. et al. Genome size analyses of Pucciniales reveal the largest fungal genomes. *Front. Plant. Sci* **5**, (2014). 10.3389/fpls.2014.00422 PMC4143883 25206357

[18] Shivas, R. G., Beasley, D. R. & McTaggart, A. R. Online identification guides for Australian smut fungi (Ustilaginomycotina) and rust fungi (Pucciniales). *IMA Fungus*. **5**, 195–202 (2014). 25734028 10.5598/imafungus.2014.05.02.03 PMC4329320

[19] Cristancho, M. A. et al. Annotation of a hybrid partial genome of the coffee rust (*Hemileia vastatrix*) contributes to the gene repertoire catalog of the Pucciniales. *Front. Plant. Sci.* **5**, (2014). 10.3389/fpls.2014.00594 PMC4215621 25400655

[20] Hawkins, N. J., Bass, C., Dixon, A. & Neve, P. The evolutionary origins of pesticide resistance. *Biol. Rev.* **94**, 135–155 (2019). 29971903 10.1111/brv.12440 PMC6378405

[21] Neve, P., Busi, R., Renton, M. & Vila-Aiub, M. M. Expanding the eco-evolutionary context of herbicide resistance research. *Pest Manag. Sci.* **70**, 1385–1393 (2014). 24723489 10.1002/ps.3757

[22] Carneiro, H. C. S., Ribeiro, N. Q., Bastos, R. W. & Santos, D. A. Effect of non-antifungal agrochemicals on the pathogenic fungus Cryptococcus gattii. *Med. Mycol.* **58**, 47–53 (2020). 30888411 10.1093/mmy/myz018

[23] Song, P. P. et al. Evaluation of antifungal activities and structure–activity relationships of coumarin derivatives. *Pest Manag. Sci.* **73**, 94–101 (2017). 27570117 10.1002/ps.4422

[24] Dai, P. et al. Synthesis, Antifungal Activity, and 3D-QASR of novel oxime ether-containing coumarin derivatives as potential fungicides. *J. Agric. Food Chem.* **72**, 5983–5992 (2024). Design. 38456397 10.1021/acs.jafc.3c06032

[25] Yildirim, M., Poyraz, S. & Ersatir, M. Recent advances on biologically active coumarin-based hybrid compounds. *Med. Chem. Res.* **32**, 617–642 (2023).

[26] Patil, S. B. Medicinal significance of novel coumarin analogs: Recent studies. *Results Chem.* **4**, 100313 (2022).

[27] Stefanachi, A. et al. A Natural, Privileged and versatile scaffold for bioactive compounds. *Molecules* **23**, 250 (2018). 29382051 10.3390/molecules23020250 PMC6017103

[28] Liu, X., Zhang, Y., Zou, Y., Yan, C. & Chen, J. Recent advances and outlook of Benzopyran derivatives in the discovery of agricultural chemicals. *J. Agric. Food Chem.* **72**, 12300–12318 (2024). 38800848 10.1021/acs.jafc.3c09244

[29] Zaynab, M., Khan, J., Al-Yahyai, R., Sadder, M. & Li, S. Toxicity of coumarins in plant defense against pathogens. *Toxicon* **250**, 108118 (2024). 39374740 10.1016/j.toxicon.2024.108118

[30] Tang, Y. et al. Recent advances of Coumarin-type compounds in discovery of pesticides. *J. Agric. Food Chem.* **72**, 26057–26073 (2024). 39557543 10.1021/acs.jafc.4c06538

[31] Guo, Y. et al. Synthesis of osthol-based botanical fungicides and their antifungal application in crop protection. *Bioorganic Medicinal Chemistry*. **40**, 116184 (2021). 33971489 10.1016/j.bmc.2021.116184

[32] Zhou, H. et al. Development of sustainable insecticide candidates for protecting pollinators: Insight into the bioactivities, selective mechanism of action and QSAR of natural coumarin derivatives against Aphids. *J. Agric. Food Chem.* **71**, 18359–18374 (2023). 37965968 10.1021/acs.jafc.3c03493

[33] Zhao, L. et al. Design, synthesis, and antiviral activities of coumarin derivatives containing dithioacetal structures. *J. Agric. Food Chem.* **68**, 975–981 (2020). 31891504 10.1021/acs.jafc.9b06861

[34] Araniti, F. et al. Phytotoxic potential and biological activity of three synthetic coumarin derivatives as new natural-like herbicides. *Molecules* **20**, 17883–17902 (2015). 26426002 10.3390/molecules201017883 PMC6331834

[35] Shi, Z., Wang, F., Zhou, W., Zhang, P. & Fan, Y. J. Application of osthol induces a resistance response against powdery mildew in pumpkin leave. *Int. J. Mol. Sci.* **8**, 1001–1012 (2007).

[36] Zhang, Z., Geng, D., Yang, Z., Pan, L. & Jin, L. Synthesis and antifungal activity of coumarin derivatives containing hydrazone moiety. *Chem. Biodivers.* **21**, e202400583 (2024). 38590217 10.1002/cbdv.202400583

[37] Yu, X. et al. Design, synthesis and antifungal activity evaluation of coumarin-3-carboxamide derivatives. *Fitoterapia* **127**, 387–395 (2018). 29631016 10.1016/j.fitote.2018.03.013

[38] Brahmachari, G. Room temperature one-pot green synthesis of coumarin-3-carboxylic acids in water: a practical method for the large-scale synthesis. *ACS Sustainable Chemistry Engineering*. **3**, 2350–2358 (2015).

[39] Vekariya, R. & Patel, H. ChemInform abstract: recent advances in the synthesis of coumarin derivatives via knoevenagel condensation: a review. *Synthetic Commun.* **44**, (2014).

[40] Koch, E. & Slusarenko, A. Arabidopsis Is susceptible to infection by a downy mildew fungus. *Plant. cell.* **2**, 437–445 (1990). 2152169 10.1105/tpc.2.5.437 PMC159900

[41] Brahmachari, G. Catalyst- and additive‐free decarboxylative C‐4 phosphorylation of coumarin‐3‐carboxylic acids at ambient conditions. *Adv. Synthesis Catalysis* **362**, (2020).

[42] Wachowska, U., Goriewa, K. & Duba, A. Charakterystyka grup fungicydów i induktorów odporności stosowanych w ograniczaniu występowania patogenów zbóż. *Zeszyty Problemowe Postępów Nauk. Rolniczych* **589**, (2017).

[43] Zhou, W. et al. Integrated pest management: an update on the sustainability approach to crop protection. *ACS Omega*. **9**, 41130–41147 (2024). 39398119 10.1021/acsomega.4c06628 PMC11465254

[44] Salehian, F. et al. A review: Biologically active 3,4-heterocycle-fused coumarins. *Eur. J. Med. Chem.* **212**, 113034 (2021). 33276991 10.1016/j.ejmech.2020.113034

[45] Moreira, M. D. et al. Compounds from Ageratum conyzoides: isolation, structural elucidation and insecticidal activity. *Pest Manag. Sci.* **63**, 615–621 (2007). 17469080 10.1002/ps.1376

[46] Xu, Z. et al. Novel jasmonic acid–coumarin pathway in the eggplant that inhibits vitellogenin gene expression to prevent mite reproduction. *J. Agric. Food Chem.* **71**, 13979–13987 (2023). 37698370 10.1021/acs.jafc.3c04007

[47] Ranjan Sahoo, C. et al. Coumarin derivatives as promising antibacterial agent(s). *Arab. J. Chem.* **14**, 102922 (2021).

[48] Zhang, M. Z., Zhang, Y., Wang, J. Q. & Zhang, W. H. Design, Synthesis and antifungal activity of Coumarin ring-opening derivatives. *Molecules* **21**, 1387 (2016). 27763520 10.3390/molecules21101387 PMC6273309

[49] Zhang, S. G. et al. Novel Coumarin 7-Carboxamide/Sulfonamide derivatives as potential fungicidal agents: design, synthesis, and biological evaluation. *Molecules* **27**, (2022). 10.3390/molecules27206904 PMC9611003 36296496

[50] Xin, W. et al. In vitro fungicidal activity and *in planta* control efficacy of coumoxystrobin against *Magnaporthe oryzae*. *Pesticide Biochem. Physiol.* 162, 78–85 (2020). 10.1016/j.pestbp.2019.09.004 31836058

[51] Al-Majedy, Y. K., Kadhum, A. A. H., Al-Amiery, A. A. & Mohamad, A. B. Coumarins: The Antimicrobial agents. *Syst. Reviews Pharm.* **8**, 62–70 (2017).

[52] Wang, Y. et al. Hydrazone derivatives in agrochemical discovery and development. *Chin. Chem. Lett.* **35**, 108207 (2024).

[53] Mapuranga, J., Zhang, N., Zhang, L., Chang, J. & Yang, W. Infection strategies and pathogenicity of biotrophic plant fungal pathogens. *Front Microbiol* **13**, (2022). 10.3389/fmicb.2022.799396 PMC9201565 35722337

[54] Lončar, M., Gašo-Sokač, D. & Molnar, M. Coumarin derivatives as antifungal agents – A review. *Czech J. Food Sci.* **41**, 79–91 (2023).

[55] Reilly, A. et al. Breadth of resistance to powdery mildew in commercial Oat cultivars available in Ireland. *Crop Prot.* **176**, 106517 (2024).

[56] Reilly, A. et al. Resistance to powdery mildew in Irish oat heritage lines. *Eur. J. Plant. Pathol.* **170**, 105–118 (2024).

[57] Jaspal, S., Shinde, V. & Kumar, A. KPF 6 -Mediated esterification and amidation of carboxylic acids. *The J. Org. Chemistry* **87**, (2022). 10.1021/acs.joc.1c02611 35060384

[58] Sonam, Shinde, V. N. & Kumar, A. KPF_6_ -Mediated esterification and amidation of carboxylic acids. *J. Org. Chem.* **87**, 2651–2661 (2022). 35060384 10.1021/acs.joc.1c02611

[59] He, X. et al. Synthesis of coumarin-3-carboxylic esters via FeCl3-catalyzed multicomponent reaction of salicylaldehydes, Meldrum’s acid and alcohols. *Tetrahedron* **71**, 863–868 (2015).

[60] Jana, R., Trivedi, R. & Tunge, J. A. Mild decarboxylative allylation of coumarins. *Org. Lett.* **11**, 3434–3436 (2009). 19588967 10.1021/ol901288r PMC2878741

[61] Szwaczko, K., Kamiński, D. M. & Koziol, A. E. Coumarin Derivatives: The Influence of Cycloalkyl Groups at the C-3 Position on Intermolecular Interactions—Synthesis, Structure and Spectroscopy. *Crystals* **14**, 196 (2024).

[62] Reilly, A. et al. Isolate-specific responses of the nonhost grass Brachypodium distachyon to the Fungal Pathogen Zymoseptoria tritici compared with Wheat. *Phytopathology* **111**, 356–368 (2021). 32720875 10.1094/PHYTO-02-20-0041-R

[63] R Core Team. ‘R: A Language and Environment for Statistical Computing’. Vienna, Austria: R Foundation for Statistical Computing. (2020). Available at: https://www.r-project.org/

