# Supplementary material for: Coumarin compounds as fungicidal agents against powdery mildew and rust in cereals
Source: Sci Rep. 2026 Feb 24;16:10385. doi: 10.1038/s41598-026-40869-w (PMC13031799; doi:10.1038/s41598-026-40869-w)
Supplement: Supplementary file 1 — Supplementary Material 1 [file 41598_2026_40869_MOESM1_ESM.docx]

| Supporting Information |
| --- |
| ***Natural Coumarin Compounds as Fungicidal Agents Against Powdery Mildew and Rust in Cereals***  **Klaudia Rząd^1*^, Aleksandra Nucia^2^, Katarzyna Szwaczko^3^, Arkadiusz Matwijczuk^1^, Sylwia Okoń^2*^**   1. Department of Biophysics, Faculty of Environmental Biology, University of Life Sciences in Lublin, Akademicka 13, 20-950 Lublin, Poland; klaudia.rzad@up.lublin.pl, arkadiusz.matwijczuk@up.lublin.pl 2. Institute of Plant Genetics, Breeding and Biotechnology, University of Life Sciences in Lublin, Akademicka 15, 20-950 Lublin, Poland; sylwia.okon@up.lublin.pl 3. Department of Organic Chemistry and Crystallochemistry, Institute of Chemical Sciences, Faculty of Chemistry, Marie Curie-Skłodowska University in Lublin, 33 Gliniana St. Lublin, Poland, katarzyna.szwaczko@umcs.pl   **Table of contents**  **1. ^1^H, ^13^C and ^31^P NMR spectra of coumarin 7, 9, 13, and 14……………………S1-S9**  **2. Statistical analysis………………………………………………………………..S10-S25** |

**^1^H, ^13^C and ^31^P NMR spectra of coumarin 7, 9, 13, and 14**

Fig.S1 ^1^H NMR of *allyl 6,8-dibromo-2-oxo-2H-chromene-3-carboxylate (****7****)*

Fig.S2 ^13^C NMR of  *allyl 6,8-dibromo-2-oxo-2H-chromene-3-carboxylate (****7****)*

Fig.S3 ^1^H NMR of  *3-chloropropyl 2-oxo-2H-chromene-3-carboxylate (****9****)*

Fig.S4 ^13^C NMR of  *3-chloropropyl 2-oxo-2H-chromene-3-carboxylate (****9****)*

Fig.S5 ^1^H NMR of  *3,7-dimethyloct-6-en-1-yl 2-oxo-2H-chromene-3-carboxylate (****13****)*

Fig.S6 ^13^C NMR of  *3,7-dimethyloct-6-en-1-yl 2-oxo-2H-chromene-3-carboxylate (****13****)*

Fig.S7 ^1^H NMR of  *(diphenylphosphoryl)methyl 2-oxo-2H-chromene-3-carboxylate (****14****)*

Fig.S8 ^13^C NMR of  *(diphenylphosphoryl)methyl 2-oxo-2H-chromene-3-carboxylate (****14****)*

Fig.S9 ^31^P NMR of  *(diphenylphosphoryl)methyl 2-oxo-2H-chromene-3-carboxylate (****14****)*

**Statistical analysis**

Fig.S10. Tukey's test for barley, variable: appressoria

Fig.S11. Tukey's test for barley, variable: haustoria

Fig.S12. Tukey's test for barley, variable: shreds

Fig.S13. Tukey's test for barley, variable: conidia

Fig.S14. Tukey's test for oat, variable: appressoria

Fig.S15. Tukey's test for oat, variable: haustoria

Fig.S16. Tukey's test for oat, variable: shreds

Fig.S17. Tukey's test for oat, variable: conidia

Fig.S18. Tukey's test for wheat, variable: appressoria

Fig.S19. Tukey's test for wheat, variable: haustoria

Fig.S20. Tukey's test for wheat, variable: shreds

Fig.S21. Tukey's test for wheat, variable: conidia

Fig.S22. Tukey's test for triticale, variable: appressoria

Fig.S23. Tukey's test for triticale, variable: haustoria

Fig.S24. Tukey's test for triticale, variable: shreds

Fig.S25. Tukey's test for triticale, variable: conidia
